# Comparative Histopathological Spectrum of Periapical Lesions in Root Canal-Treated and Non-root Canal-Treated Teeth

**DOI:** 10.7759/cureus.115873

**Published:** 2026-09-06

**Authors:** Sachin Kandalkar, Sadhvi Gupta, Chidipothu Sri Madhavi, Abhishek Vilekar, Shilpa Kuravatti, Pradnya Relan, Satyabrat Banerjee

**Affiliations:** 1 Department of Oral Pathology, Sau Mathurabai Bhausaheb Thorat Dental College and Hospital, Sangamner, IND; 2 Department of Conservative Dentistry and Endodontics, Baba Jaswant Singh Dental College, Hospital and Research Institute, Ludhiana, IND; 3 Department of Pedodontics and Preventive Dentistry, Mamata Institute of Dental Sciences, Hyderabad, IND; 4 Department of Prosthodontics, Sree Sai Dental College and Research Institute, Srikakulam, IND; 5 Department of Oral Medicine and Radiology, Tatyasaheb Kore Dental College and Research Centre, New Pargaon, IND; 6 Department of Oral Pathology and Microbiology, Jawahar Medical Foundation's Annasaheb Chudaman Patil Memorial Dental College, Dhule, IND; 7 Department of Conservative Dentistry and Endodontics, Jawahar Medical Foundation's Annasaheb Chudaman Patil Memorial Dental College, Dhule, IND

**Keywords:** histopathology, periapical abscess, periapical granuloma, periapical lesion, radicular cyst, root canal treatment

## Abstract

Introduction: Periapical lesions represent a spectrum of inflammatory and pathological changes associated with endodontic infection. The histopathological characteristics may differ according to the previous root canal treatment status of the associated tooth. This study aimed to compare the histopathological spectrum and predominant inflammatory patterns of periapical lesions in root canal-treated (RCT) and non-root canal-treated (non-RCT) teeth.

Materials and methods: A comparative cross-sectional histopathological study was conducted on 120 periapical tissue specimens comprising 60 RCT and 60 non-RCT specimens. Specimens were obtained during periapical surgery or extraction and examined histopathologically using hematoxylin and eosin staining. The histopathological diagnoses were categorized as periapical granuloma, radicular cyst, periapical abscess, or other/indeterminate. Predominant inflammation was classified as chronic, acute, or mixed acute-on-chronic inflammation. Two blinded oral and maxillofacial pathologists independently evaluated the specimens, and interobserver agreement was assessed using Cohen's kappa. Categorical variables were analyzed using chi-squared or Fisher's exact tests, with odds ratios (ORs) and 95% confidence intervals (CIs) calculated for the individual diagnostic categories.

Results: The mean age was 36.67±10.18 years in the RCT group and 36.86±10.00 years in the non-RCT group (p=0.917). Periapical granuloma occurred in 20 (33.3%) RCT and 32 (53.3%) non-RCT specimens (OR=0.44; 95% CI: 0.21-0.92; p=0.042), whereas radicular cysts occurred in 28 (46.7%) and 14 (23.3%), respectively (OR=2.88; 95% CI: 1.31-6.30; p=0.012). Periapical abscess occurred in eight (13.3%) RCT and 10 (16.7%) non-RCT specimens (OR=0.77; 95% CI: 0.28-2.11; p=0.799). Predominantly, chronic inflammation was observed in 34 (56.7%) RCT and 22 (36.7%) non-RCT specimens, whereas acute inflammation occurred in 8 (13.3%) and 20 (33.3%) specimens, respectively (χ²=7.71; p=0.021). Interobserver agreement was almost perfect for histopathological diagnosis (κ=0.874) and substantial for inflammatory patterns (κ=0.805).

Conclusion: Periapical lesions associated with RCT and non-RCT teeth demonstrated significant differences in histopathological diagnosis and inflammatory patterns. Radicular cysts were more frequent in RCT teeth, whereas granulomas and acute inflammation were more frequent in non-RCT teeth than in RCT teeth. Histopathological assessment provides valuable diagnostic information regarding persistent periapical lesions.

## Introduction

Periapical lesions represent a common consequence of pulpal infection and are characterized by inflammatory and immunological responses in periapical tissues [1]. The progression of microbial infection from the root canal system to the periapical tissues may result in a spectrum of lesions ranging from chronic inflammatory lesions such as periapical granulomas to radicular cysts and acute suppurative lesions such as periapical abscesses [2]. Although these lesions may exhibit similar clinical and radiographic features, their underlying histopathological characteristics can vary considerably [3]. Therefore, histopathological examination remains an important method for establishing the definitive nature of periapical lesions, particularly when clinical and radiographic findings are inconclusive [4].

Root canal treatment is performed to eliminate intracanal infection and prevent or resolve periapical inflammation [5]. However, persistent periapical lesions may occur following endodontic treatment because of residual or persistent microorganisms, inadequate disinfection, missed anatomy, reinfection, or extraradicular infection [6]. Consequently, periapical lesions associated with root canal-treated (RCT) teeth may demonstrate a different inflammatory and histopathological profile from those associated with teeth that have not previously undergone root canal treatment [7]. A comparison of these lesions may provide insights into the biological responses associated with persistent periapical disease following endodontic interventions.

The present study was designed to compare the histopathological characteristics of periapical lesions between RCT and non-root canal-treated (non-RCT) teeth. The primary aim was to evaluate whether the distribution of major histopathological diagnoses differed according to previous root canal treatment status. The secondary objective was to compare the predominant inflammatory patterns, including chronic, acute, and mixed acute-on-chronic inflammation, between the two groups. By correlating histopathological findings with endodontic treatment status, this study sought to provide a clearer understanding of the pathological spectrum of periapical lesions and the tissue response associated with persistent periapical disease.

## Materials and methods

Study design and setting

A comparative cross-sectional histopathological study was conducted in the Department of Oral Pathology and Microbiology in collaboration with the Department of Conservative Dentistry and Endodontics, Jawahar Medical Foundation's Annasaheb Chudaman Patil Memorial Dental College, Dhule, India, between September 2025 and March 2026. The study protocol was reviewed and approved by the institute's Institutional Ethics Committee (approval number: EC/NEW/INST/2022/2959/2025/08/SS2; date: August 25, 2025) before the commencement of the study. This study was conducted in accordance with the ethical principles of the Declaration of Helsinki, and written informed consent was obtained from all participants for the use of clinically indicated surgical or extraction specimens for research purposes. Patient identifiers were removed from the study database, and all clinical and histopathological records were maintained.

Study population and group allocation

The study included patient data of permanent anterior and posterior teeth with radiographically evident periapical lesions. Patients were eligible when a localized periapical radiolucency was present, and representative periapical tissue was obtained during periapical surgery or extraction and submitted for histopathological examination. Based on the previous endodontic treatment status, the specimen data were categorized into two groups: Group I comprised periapical lesions associated with RCT teeth, whereas Group II comprised lesions associated with non-RCT teeth. Patients with inadequate or non-representative tissue, extensive cautery or processing artifacts, uncertain endodontic treatment status, primary teeth, developmental cysts or tumors unrelated to endodontic infection, or inadequate clinical or radiographic documentation were excluded.

Sample size and sampling

The sample size was calculated a priori using G*Power, Version 3.1.9.7 (Heinrich Heine University Düsseldorf, Düsseldorf, Germany), based on an anticipated effect size of 0.6, a two-sided significance level of 5%, and a 90% statistical power for an independent-samples t-test [8]. The initial calculation indicated a requirement of 54 specimens per group. To account for a potential 10% rate of inadequate or noninterpretable specimens, this number was inflated to 60 specimens per group. The study finally comprised 120 specimens, with 60 specimens allocated to each study group.

Clinical and radiographic assessment

Clinical and radiographic information obtained at the time of specimen collection was also considered. The previous root canal treatment status was established based on the clinical history and confirmed by intraoral radiographic examination. The principal clinical variable was categorized as RCT or non-RCT status, whereas the principal radiographic finding was periapical radiolucency. Where available, lesion size was recorded from standardized periapical radiographs and was considered a descriptive covariate rather than a primary outcome. Lesion size was measured on standardized periapical radiographs using a digital caliper within radiography software (ImageJ, Version 1.53, National Institutes of Health, Bethesda, Maryland, United States). The maximum mesiodistal and superoinferior dimensions were recorded, and the mean of these two measurements was calculated to account for irregular lesion morphology. Lesions with regular morphology were measured by their maximum diameter. Measurements were recorded in millimeters (mm). Radiographic data adequate for size measurement were available for 92 of the 120 specimens (76.7%); the remaining 28 cases (23.3%) lacked sufficient radiographic quality or were unavailable at the time of specimen collection. Given that lesion size was a descriptive covariate and not a primary outcome, cases with missing measurements were excluded from the descriptive size analysis only and retained in all primary analyses, consistent with the missing-at-random assumption.

Specimen collection and histopathological processing

Periapical tissue specimens were obtained during clinically indicated periapical surgical procedures or extractions using standard aseptic techniques. During periapical surgery, the periapical tissue was carefully curetted using a Lucas surgical curette, and the excised tissue was retrieved using sterile tissue forceps. In cases of extraction, the periapical tissue associated with the extracted tooth was similarly collected using sterile surgical instruments. The specimens were immediately transferred to labeled specimen containers containing 10% neutral buffered formalin (Chempure, Chempure Pvt. Ltd., Bengaluru, India) for fixation. Following fixation, the specimens were subjected to routine tissue processing, including dehydration, clearing, and conventional paraffin embedding. The processed tissues were embedded in paraffin wax, and serial sections approximately 3-5 μm in thickness were prepared using a rotary microtome (RMT-45, Radical Scientific Pvt. Ltd., Ambala, India). The sections were mounted on glass slides and stained with hematoxylin and eosin (H&E) (Merck, Merck Life Science Pvt. Ltd., Mumbai, India). The stained sections were examined microscopically using a Labomed Lx200 microscope (Labomed, Gurugram, India). Special stains and immunohistochemical investigations were not routinely performed because the histopathological assessment was based on conventional H&E morphology.

Histopathological assessment

H&E-stained sections were independently examined by two experienced oral and maxillofacial pathologists (SK and PR). Examiners were blinded to the endodontic treatment status of the corresponding teeth during the initial assessment. Each examiner independently recorded the findings using a standardized assessment form before any discussion between the examiners. The primary histopathological diagnoses were classified as periapical granuloma, radicular cyst, periapical abscess, or other/indeterminate. A periapical granuloma was identified by granulation tissue containing predominantly chronic inflammatory cells, proliferating fibroblasts, small blood vessels, and variable inflammatory cell infiltration, without a well-defined epithelial cavity. A radicular cyst was identified as a pathological cavity lined with stratified squamous epithelium within an inflamed fibrous connective tissue wall. A periapical abscess was identified by the localized accumulation of neutrophils, with tissue destruction consistent with suppurative inflammation. Cases that could not be confidently assigned to a specific category were recorded as other or indeterminate (Figure 1).

**Figure 1:**
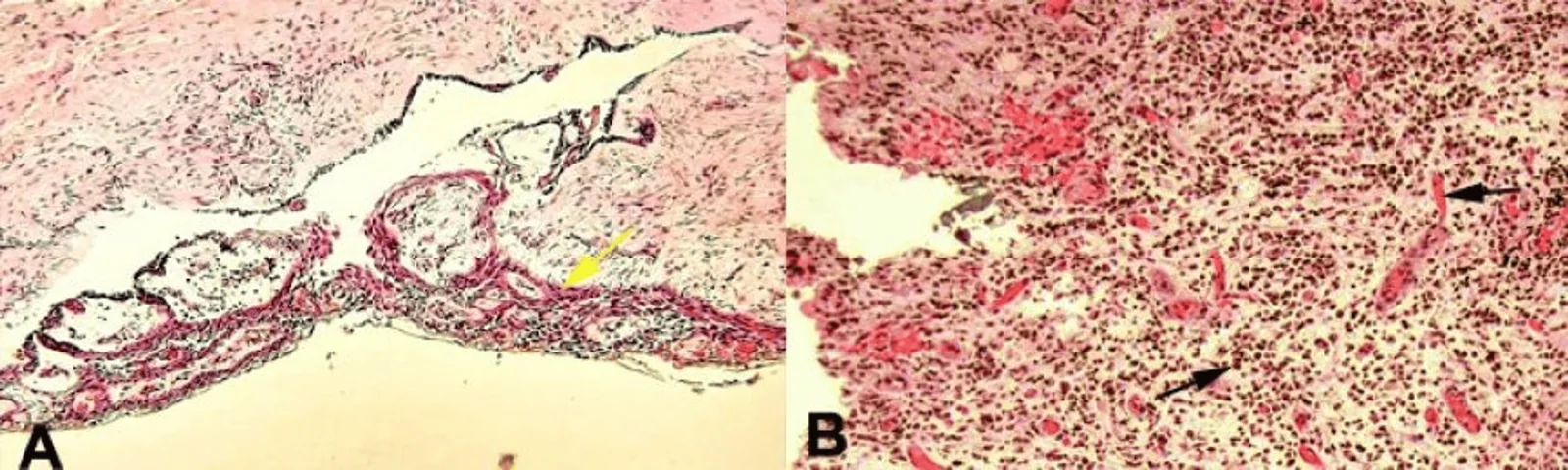
Images from the case studies (A) Hematoxylin and eosin-stained section of a periapical cyst showing epithelial lining (yellow arrow) at 10× magnification. (B) Hematoxylin and eosin-stained section of a periapical granuloma showing inflammatory cells (black arrows) at 40× magnification.

The predominant inflammatory pattern was categorized as predominantly chronic, acute, or mixed acute-on-chronic. Chronic inflammation is predominantly characterized by the presence of lymphocytes, plasma cells, and macrophages, whereas acute inflammation is predominantly characterized by neutrophilic infiltration. A mixed pattern was recorded when substantial acute and chronic inflammatory components were simultaneously present. No semiquantitative scoring of the individual inflammatory cell populations was performed.

Calibration, blinding, and interobserver agreement

Before formal assessment, both pathologists were standardized according to predefined diagnostic categories and inflammatory pattern criteria. The same assessment criteria and standardized recording form were used for all specimens. During the initial microscopic evaluation, the examiners were blinded to the RCT status to minimize observer and classification bias. In cases of disagreement, the sections were reviewed jointly after independent assessments were recorded and a consensus diagnosis was established. Interobserver agreement was assessed using Cohen's kappa coefficient (κ). For the primary histopathological diagnosis, the observed agreement was 91.7% (κ=0.874), whereas the predominant inflammatory pattern demonstrated 87.5% agreement (κ=0.805), indicating substantial-to-almost-perfect agreement.

Statistical analysis

Statistical analyses were performed using IBM SPSS Statistics for Windows, Version 28.0 (IBM Corp., Armonk, New York, United States). Continuous variables were expressed as mean±standard deviation, while categorical variables were presented as frequencies and percentages. Baseline age was compared using the independent-samples t-test, and sex was compared using Pearson's chi-squared test. The distribution of histopathological diagnoses between the RCT and non-RCT groups was assessed using Pearson's chi-squared test. When the expected cell frequencies were inadequate, clinically appropriate categories were combined before the analysis. Cramér's V has been reported to be a measure of association. Predominant inflammatory patterns were compared using Pearson's chi-squared test. Category-specific associations were further evaluated using odds ratios (ORs) with 95% confidence intervals (CIs), and Fisher's exact test was used for 2×2 comparisons. Interobserver agreement was assessed using Cohen's kappa statistic. All tests were two-sided, and statistical significance was set at p<0.05.

## Results

Baseline characteristics of the study groups

A total of 120 periapical tissue specimens were included in the analysis, comprising 60 specimens from extracted RCT teeth and 60 from non-RCT teeth. The mean age was 36.67±10.18 years in the RCT group and 36.86±10.00 years in the non-RCT group, with no significant difference between the groups (p=0.917). Similarly, the sex distribution did not differ significantly between the RCT and non-RCT groups (p=0.583). The baseline characteristics of the study groups are presented in Table 1.

**Table 1:** Baseline characteristics of the study groups Data are presented as mean±standard deviation (SD) for continuous variables and n (%) for categorical variables. Between-group comparisons were performed using the independent-samples t-test for age and Pearson's chi-squared test for sex. p<0.05 was considered statistically significant. d: Cohen's d; φ: phi coefficient

Characteristic	Root canal-treated group (n=60)	Non-root canal-treated group (n=60)	Test statistic	P-value	Effect size
Age, years (mean±SD)	36.67±10.18	36.86±10.00	t=-0.1	0.917	d=-0.02
Sex					
Male, n (%)	34 (56.7%)	31 (51.7%)	χ²=0.3	0.583	φ=0.05
Female, n (%)	26 (43.3%)	29 (48.3%)			

Distribution of histopathological diagnoses

The distribution of histopathological diagnoses differed between the two groups. Periapical granulomas were identified in 20 (33.3%) specimens in the RCT group compared with 32 (53.3%) in the non-RCT group, whereas radicular cysts were observed in 28 (46.7%) and 14 (23.3%) specimens, respectively. Periapical abscesses were identified in 8 (13.3%) RCT specimens and 10 (16.7%) non-RCT specimens, while other or indeterminate lesions accounted for 4 (6.7%) specimens in each group. This analysis demonstrated a significant difference in the distribution of histopathological diagnoses between the groups (χ²=7.59; df=3; p=0.022; Cramér's V=0.251) (Table 2).

**Table 2:** Distribution of primary histopathological diagnoses by study group Data are presented as n (%). Pearson's chi-squared test was used for comparison between the groups. *p<0.05 was considered statistically significant. df: degrees of freedom

Histopathological diagnosis	Root canal-treated group n (%)	Non-root canal-treated group n (%)	Test value (df)	P-value	Effect size (Cramer's V)
Periapical granuloma	20 (33.3%)	32 (53.3%)	χ²=7.59 (3)	0.022*	0.251
Radicular cyst	28 (46.7%)	14 (23.3%)			
Periapical abscess	8 (13.3%)	10 (16.7%)			
Other/indeterminate	4 (6.7%)	4 (6.7%)			

Predominant inflammatory patterns

The predominant inflammatory pattern also differed significantly between the groups. Chronic inflammation was predominantly observed in 34 (56.7%) and 22 (36.7%) specimens from the RCT and non-RCT groups, respectively. In contrast, predominantly acute inflammation was present in 8 (13.3%) RCT specimens compared with 20 (33.3%) non-RCT specimens. A mixed acute-on-chronic inflammatory pattern was identified in 18 (30%) specimens from each group. The difference in inflammatory pattern distribution was statistically significant (χ²=7.71; df=2; p=0.021; Cramér's V=0.254) (Table 3).

**Table 3:** Distribution of predominant inflammatory patterns in the root canal-treated and non-root canal-treated groups Data are presented as n (%). Differences between the groups were assessed using Pearson's chi-squared test. *p<0.05 was considered statistically significant. df: degrees of freedom

Predominant inflammatory pattern	Root canal-treated group n (%)	Non-root canal-treated group n (%)	Test value (df)	P-value	Effect size (Cramer's V)
Predominantly chronic	34 (56.7%)	22 (36.7%)	χ²=7.71 (2)	0.021*	0.254
Predominantly acute	8 (13.3%)	20 (33.3%)			
Mixed acute-on-chronic	18 (30%)	18 (30%)			

Odds ratios for individual histopathological diagnoses

Category-specific analysis showed that RCT status was associated with lower odds of periapical granuloma than non-RCT status (OR=0.44; 95% CI: 0.21-0.92; p=0.042). Conversely, radicular cysts were associated with higher odds ratios in the RCT group (OR=2.88; 95% CI: 1.31-6.30; p=0.012). No significant association was observed for periapical abscesses (OR=0.77; 95% CI: 0.28-2.11; p=0.799) or other/indeterminate lesions (OR=1.00; 95% CI: 0.24-4.20; p=1.000) (Table 4).

**Table 4:** Odds ratios for individual histopathological diagnoses comparing the root canal-treated and non-root canal-treated groups Data are presented as n=60. Category-specific associations were assessed using Fisher's exact test. Odds ratios and 95% confidence intervals (CIs) were calculated for each histopathological diagnosis compared with all other diagnoses. *p<0.05 was considered statistically significant.

Diagnosis	Root canal-treated group (n=60)	Non-root canal-treated group (n=60)	Odds ratio	95% CI	P-value
Periapical granuloma	20 (33.3%)	32 (53.3%)	0.44	0.21-0.92	0.042*
Radicular cyst	28 (46.7%)	14 (23.3%)	2.88	1.31-6.30	0.012*
Periapical abscess	8 (13.3%)	10 (16.7%)	0.77	0.28-2.11	0.799
Other/indeterminate	4 (6.7%)	4 (6.7%)	1.00	0.24-4.20	1.000

Reference category and outcome definitions are presented in Table 5.

**Table 5:** Reference category and outcome definition for each OR OR: odds ratio

Diagnosis	Outcome definition	Grouping variable	Reference category	Interpretation of OR
Periapical granuloma	Presence of granuloma vs. all other diagnoses (radicular cyst, abscess, other/indeterminate)	Root canal-treated vs. non-root canal-treated	Non-root canal-treated group	OR=0.44 indicates that root canal-treated teeth have 56% lower odds of being diagnosed as granuloma compared to non-root canal-treated teeth
Radicular cyst	Presence of radicular cyst vs. all other diagnoses (granuloma, abscess, other/indeterminate)	Root canal-treated vs. non-root canal-treated	Non-root canal-treated group	OR=2.88 indicates that root canal-treated teeth have 2.88 times higher odds of being diagnosed as radicular cyst compared to non-root canal-treated teeth
Periapical abscess	Presence of abscess vs. all other diagnoses (granuloma, cyst, other/indeterminate)	Root canal-treated vs. non-root canal-treated	Non-root canal-treated group	OR=0.77 indicates that root canal-treated teeth have 23% lower odds of being diagnosed as abscess compared to non-root canal-treated teeth (not significant)
Other/indeterminate	Presence of other/indeterminate diagnosis vs. all other diagnoses (granuloma, cyst, abscess)	Root canal-treated vs. non-root canal-treated	Non-root canal-treated group	OR=1.00 indicates no difference in the odds of indeterminate diagnosis between the groups

Interobserver agreement

The interobserver agreement between the two pathologists was high. For the primary histopathological diagnosis, the observed agreement was 91.7% with a Cohen's κ value of 0.874. For the predominant inflammatory pattern, the observed agreement was 87.5% with a Cohen's κ value of 0.805 (Table 6).

**Table 6:** Interobserver agreement for histopathological assessment between the two pathologists Data are presented as number of paired readings, observed agreement (%), and Cohen's kappa coefficient (κ). Interobserver agreement was assessed using Cohen's kappa statistic. κ: Cohen's kappa coefficient

Outcome assessed	N (paired readings)	Observed agreement	Cohen's κ
Primary histopathological diagnosis	120	91.7%	0.874
Predominant inflammatory pattern	120	87.5%	0.805

## Discussion

The present study demonstrated a distinct histopathological distribution of periapical lesions according to previous root canal treatment status. Although the four-category comparison showed a borderline overall difference, after combining the categories with low expected frequencies, the analysis demonstrated a statistically significant difference between the RCT and non-RCT groups. The most notable finding was the greater proportion of radicular cysts in RCT teeth compared to non-RCT teeth, whereas periapical granulomas were more frequent in non-RCT teeth than in RCT teeth. The category-specific analysis supported these observations, showing significantly lower odds of granulomas in the RCT group and significantly higher odds of radicular cysts.

The higher frequency of radicular cysts among previously treated teeth may reflect the persistence of lesions that were already established before endodontic treatment or the persistence of infection capable of maintaining an epithelialized periapical lesion despite treatment [9]. Persistent apical periodontitis after root canal treatment can be associated with residual or persistent intraradicular microorganisms, extraradicular infection, inadequate disinfection, and other factors that allow inflammation to persist [6]. Nair's review also emphasized that persistent apical disease may have complex microbial and pathological mechanisms and that the histopathological distinction of cystic lesions is particularly dependent on adequate tissue sampling and sectioning [6]. Importantly, the present cross-sectional design cannot establish whether the cysts developed after RCT or were pre-existing lesions that persisted despite treatment.

The higher frequency of radicular cysts observed in the RCT group in the present study is consistent with previous histopathological investigations of persistent periapical lesions in the root-filled teeth. Love and Firth [10] examined 100 surgically removed persistent periapical lesions and reported periapical granuloma as the most common diagnosis, with cysts accounting for 18% of cases. Their findings confirmed that both granulomatous and cystic lesions are important pathological presentations of persistent periapical disease following root canal treatment.

The predominance of granulomas in non-RCT teeth is biologically plausible because untreated infected canals can produce a continuous microbial stimulus that induces a chronic inflammatory response in the periapical tissues [11]. Therefore, granulation tissue may represent an active host response that attempts to contain infection. A previous study consistently identified granuloma as one of the most frequent forms of periapical pathology [8]. The reported histopathological prevalence of periapical granulomas and radicular cysts varies considerably among studies, likely reflecting differences in case selection, specimen collection, and histopathological sampling methods. Omoregie et al. [12] reported granulomas in 16.1% and cysts in 1.5% of 75 chronic periapical lesions, illustrating the substantial variability in the reported distribution of these lesions.

The inflammatory pattern findings further support the difference in the pathological environment between the two groups. Predominantly, chronic inflammation is more common in RCT teeth, whereas acute inflammation is more frequent in non-RCT teeth. Overall, the differences were statistically significant. This may indicate that lesions associated with previously treated teeth in this sample were more likely to represent longstanding and persistent inflammatory processes, whereas untreated teeth may retain a more active inflammatory stimulus. Nevertheless, this interpretation should be cautious because the treatment duration, quality of previous root canal treatment, time since treatment, microbial status, and lesion duration were not evaluated.

The present findings also reinforce the importance of histopathological examination in the definitive characterization of periapical lesions [13]. However, clinical and radiographic findings alone cannot reliably distinguish between granulomas and cysts. In one study of surgically treated periapical lesions, histopathological examination identified 34 granulomas and 18 radicular cysts, whereas clinical/radiographic assessments showed discrepancies in individual cases [14]. The distribution of periapical lesions varies considerably among populations and study settings. In a large Brazilian multicenter study of 10,381 histopathologically diagnosed chronic inflammatory periapical lesions, radicular cysts were the most frequent lesion, accounting for 59.9% of cases, whereas periapical granulomas and abscesses constituted the remaining lesions [15]. This variation compared with the present findings may be related to differences in case selection, treatment status, referral patterns, and histopathological sampling.

From a clinical perspective, these findings suggest that persistent periapical lesions associated with previously RCT teeth warrant careful evaluation rather than being assumed to represent simple recurrent inflammation. Histopathological examination of tissues obtained during periapical surgery or extraction can provide definitive diagnostic information and may assist in distinguishing persistent cystic pathologies from granulomatous or suppurative lesions. This distinction may be particularly relevant when treatment decisions involve non-surgical retreatment versus surgical intervention.

This study had several limitations. Its cross-sectional design prevents the determination of the temporal relationships between root canal treatment and the development or persistence of histopathological lesions. This study was conducted at a single institution with a relatively modest sample size of 120 specimens, which may limit the generalizability of the results. Information regarding the quality and technical characteristics of previous root canal treatment, duration since treatment, microbial status, lesion duration, and lesion size is limited. Furthermore, conventional H&E examination was performed without routine special staining or immunohistochemical characterization, which restricts the assessment of morphological features. Finally, although examiner blinding and high interobserver agreement strengthened the diagnostic reliability, histopathological classification remained dependent on tissue sampling and the representativeness of the submitted specimen.

## Conclusions

Within the limitations of this study, periapical lesions associated with RCT and non-RCT teeth demonstrated significant differences in histopathological diagnosis and inflammatory patterns. Radicular cysts were more frequently associated with RCT teeth, whereas periapical granulomas were more common in non-RCT teeth. Chronic inflammation was predominant in the RCT group, whereas acute inflammation was more frequent in the non-RCT group. Therefore, histopathological examination remains valuable for the definitive characterization of persistent periapical lesions.
